# 套细胞淋巴瘤诊断与治疗中国指南（2022年版）解读

**DOI:** 10.3760/cma.j.issn.0253-2727.2022.11.004

**Published:** 2022-11

**Authors:** 禹廷 阎, 树华 易, 录贵 邱

套细胞淋巴瘤（mantle cell lymphoma, MCL）是一种起源于成熟B细胞的非霍奇金淋巴瘤亚类，占非霍奇金淋巴瘤（NHL）的6％～8％[1]。近年来，MCL的基础转化研究与临床治疗均取得重大进展，中国抗癌协会血液肿瘤专业委员会、中华医学会血液学分会和中国临床肿瘤学会淋巴瘤专家委员会共同制定了《套细胞淋巴瘤诊断与治疗中国指南（2022年版）》（以下简称“指南”）[2]，本文将针对MCL诊疗过程中的重点、难点及争议问题进行讨论，对本指南的更新部分进行解读，同时结合国内外最新研究进展对指南相关推荐进行补充和拓展，为临床实践提供更多参考。

一、诊断

MCL的诊断需满足以下三条中的任意一条：

（1）典型的组织形态学特征联合成熟B细胞免疫特征，再加上免疫组化CD5和Cyclin D1核内阳性。

（2）如果无法进行组织学检查，骨髓或外周血肿瘤细胞免疫表型需符合典型MCL，且常规染色体核型检出t（11;14）或荧光原位杂交（FISH）检出CCND1/IGH。

（3）组织形态学特征和免疫表型符合典型MCL，但Cyclin D1和t（11;14）均阴性，则免疫组化检测SOX11，如果SOX11阳性，亦可诊断MCL，有条件可以加做FISH检测CCND2或CCND3重排。

t（11;14）或Cyclin D1过表达在成熟B细胞淋巴瘤中对诊断MCL具有特异性，浆细胞瘤可出现t（11;14）和Cyclin D1阳性，此外，毛细胞白血病患者也可出现Cyclin D1弱阳性，5％左右弥漫大B细胞淋巴瘤（DLBCL）可出现Cyclin D1阳性，但通常缺乏t（11;14），若出现t（11;14），应诊断为MCL，特别是在双打击高级别B细胞淋巴瘤中，若伴有CCND1重排，WHO分类2016版建议首先诊断为MCL。部分淋巴瘤在复发后可能出现继发t（11;14），此时不诊断为MCL，而应诊断为原发疾病。

部分患者会表现为非典型的免疫表型或免疫组化特征，在诊断MCL过程中，我们通常要求CD5、CCND1和SOX11三个指标中至少有两个阳性，并结合其他组织学和免疫表型特征鉴别诊断。但并非所有CCND1高表达的MCL均伴有t（11;14），有少数患者存在累及免疫球蛋白轻链位点的变异易位，如t（11;22）（q13;q11）/CCND1-IGL和t（2;11）（p11;q13）/IGK-CCND1[3]–[4]。另有约5％的MCL患者为CCND1阴性，可伴有CCND2/CCND3的高表达，细胞遗传学异常主要表现为CCND2/CCND3与免疫球蛋白轻链的重排（IGK/CCND2易位最多见），另有极少数表现为CCNE1或CCNE2的高表达[5]。研究发现，CCND1阴性和CCND1阳性的MCL患者表现出相似的基因组学特征和临床预后[6]，对于CCND1阴性的患者，免疫组化SOX11的检测对鉴别诊断有重要提示意义，加做CCND2/CCND3断裂探针的FISH检测也有重要的辅助诊断意义。

诊断MCL应进行形态学描述，10％～15％的MCL细胞形态呈“侵袭性变型”，侵袭性变型可分为母细胞变型和多形性变型，预后差。

二、分型

MCL临床异质性强，在诊断后可以分为两型，第一种为经典型MCL（classic MCL，cMCL），占MCL的绝大部分，生物学行为多样；第二种为白血病型非淋巴结型MCL（non-nodal MCL，nnMCL），多数临床呈惰性表现，研究发现cMCL和nnMCL有不同的基因突变谱，并且肿瘤细胞起源、信号通路调控及表观遗传学异常均存在差异[7]–[8]。国内外MCL全外显子测序研究均发现，nnMCL TP53突变/缺失的比例较高（30％～40％），且伴有TP53异常的nnMCL仍可呈惰性病程，生存时间较长[8]–[9]，因此在更新的指南中，TP53异常不再作为cMCL和nnMCL的鉴别要点。需要强调的是指南中nnMCL诊断的建议条件并非金标准，需要结合患者的临床和生物学特征进行综合诊断。

更新后的指南将原位套细胞肿瘤（ISMCN）归为MCL的一个特殊亚型，ISMCN Cyclin D1阳性的B细胞仅局限分布于淋巴滤泡套区，但未破坏淋巴结结构，这部分患者极少进展，预后好[10]。

三、预后评估

建立精准的预后评估体系是实现分层治疗和个体化治疗的基础。MCL国际预后评分系统（MIPI评分）通过年龄、ECOG体力状况、乳酸脱氢酶和WBC四个指标对MCL患者进行预后分层[11]。随后的研究发现，将Ki-67指数加入MIPI评分系统（MIPI-c）可以进一步将患者分为危险度不同的四组，更好地鉴别出高危人群[12]。上述两种评分系统主要基于患者的临床特征，其他生物学预后指标，如侵袭性变型、TP53突变/缺失、MYC扩增/易位、CDKN2A（9p）缺失、复杂核型等，都是免疫化疗时代MCL较明确的不良预后因素。另外随着新型分子靶向药的使用，一些特殊的遗传学异常可能与靶向药耐药相关，如SMARCA4突变、9p缺失、BCL2L1上调可能与伊布替尼及维奈克拉耐药相关[13]，CARD11突变和BIRC3突变可能与伊布替尼耐药相关[14]，BCL2扩增子缺失可能与维奈克拉耐药相关[15]（表1）。MCL的临床及遗传学特征存在高度异质性，患者对治疗的反应及预后也相差很大，我们前期建立的基于遗传学特点的分子分型将有助于临床危险度分层并指导精准治疗[8]。

**表1:** 套细胞淋巴瘤（MCL）的预后因素总结

已纳入临床常规应用的预后因素	目前证据较明确的预后因素	证据不足的预后因素
年龄、ECOG评分、血清LDH、WBC、Ki-67指数、临床分期（Ann Arbor分期）、β_2_-微球蛋白、病理（经典型对母细胞变型）、临床分型（cMCL对nnMCL）	IGHV突变状态、TP53突变/缺失、CDKN2A（9p）缺失、MYC扩增/易位、复杂核型	MCL35 RNA基因表达评分、BCR通路基因表达、NOTCH1突变、KMT2D突变、SMARCA4突变、CARD11突变、TRAF2突变、BIRC3突变、截短的CCND1突变

注 cMCL：经典型MCL；nnMCL：白血病型非淋巴结型MCL

四、治疗

大多数MCL患者呈侵袭性病程，10％～15％的患者表现为惰性病程，不局限于nnMCL。惰性MCL早期干预并没有改善患者的总生存（OS），因此指南推荐无治疗指征的患者可观察等待，治疗指征的评估可参考CLL，超过半数患者在观察的前两年不需要治疗[16]，待出现治疗指征后，可参照如下治疗策略。

1. 早期患者的治疗：大多数cMCL在诊断时即为晚期，且多伴有骨髓、肝脾和胃肠道等结外侵犯，但仍有10％～15％的患者诊断为早期局限型MCL（Ann Arbor Ⅰ～Ⅱ期），这部分患者需要常规化疗还是强化化疗目前还存在争议。韩国的一项多中心研究对41例早期患者进行常规化疗[17]，另一项国际淋巴瘤放射肿瘤学组的研究总结了应用不同方案治疗179例早期MCL患者的长期随访数据[18]，这部分早期局限型MCL患者大多年龄较高，耐受性差，无论是否应用强化方案，患者的OS相似[19]。如何应用低毒性方案实现长期控制是此部分患者的治疗目标，有研究认为单纯放疗可能会导致早期复发的风险增加[20]，目前ESMO和NCCN指南均推荐，免疫化疗（非强化方案）±受累野放疗是这部分患者的较优选择。但伴有大肿块病变（>5 cm）和其他高危因素的患者应用非强化方案预后较差[21]，因此，对于伴有高危因素的Ⅰ～Ⅱ期患者，指南建议按照晚期（Ⅲ～Ⅳ期）进行治疗。高危因素包括：大肿块病变（≥5 cm）、Ki-67>30％、TP53突变/缺失、细胞形态为侵袭性变型等。

2. 晚期患者的治疗：鉴于TP53突变患者常规治疗效果极差，中位生存时间不足2年，NCCN指南将晚期（Ⅲ～Ⅳ期）MCL伴TP53突变患者单列出来，强调对其进行新的治疗方案的探索。本版指南中，我们扩大了高危组的范围，将标准治疗下OS时间不足2～3年的人群均纳入高危组范围，包括TP53突变、TP53和CDNK2A缺失、侵袭性变型、MIPI-c高危组[8],[12],[22]–[23]。目前对高危组患者如何进行治疗处于探索中，新药的加入是探索的方向，但已有的证据表明，一线治疗中单纯加入BTK抑制剂不能克服或改善这些患者的预后，多种新药组合可能是方向，如BTK抑制剂联合BCL2抑制剂等。

对于非高危且年轻（年龄≤65岁）、一般情况较好的患者，利妥昔单抗联合中大剂量的阿糖胞苷（Ara-C）诱导化疗后续贯ASCT仍是一线推荐。一项欧洲随机对照研究发现，与单独R-CHOP诱导组相比，R-CHOP/DHAP诱导组的中位无进展生存（PFS）时间明显延长（9.1年对3.9年，*P*＝0.038）[24]。美国M.D. Anderson癌症中心的一项研究采用R-Hyper CVAD/R-MA交替后不进行移植，完全缓解（CR）率高达87％，长期随访中位OS时间达10.8年[25]，但需要注意的是此方案不良反应发生率较高，二次肿瘤发生率较高（26％）[26]。

年轻MCL治疗的另一个问题是对于非高危组患者，联合新药治疗是否可以取代ASCT。WINDOW-1临床试验[27]可以部分回答这个问题，该研究前期采用IR诱导，最多12个周期，后续依据不同治疗反应给予R-HyperCVAD/R-MA化疗4～8个疗程，不进行ASCT和利妥昔单抗维持，131例MCL患者纳入研究，80％患者为MIPI低危组。初步结果发现，伊布替尼+利妥昔单抗诱导方案的反应率较高［总反应率（ORR）98％，CR率87％］，缓解率与既往大剂量阿糖胞苷为基础的诱导治疗相近。中位随访42个月，所有患者3年PFS率79％，OS率95％，低危组患者预后更佳，因此推测年轻低危患者在加入BTK抑制剂的情况下可能不需要ASCT。而最终回答这一问题需要等待欧洲大型Ⅲ期随机对照TRIANGLE试验结果，该试验将患者随机分为三组，标准治疗组：R-CHOP/R-DHAP交替后行ASCT巩固，后予以利妥昔单抗维持；试验组1：标准治疗组中加入伊布替尼；试验组2：在试验组1的基础上去掉ASCT。该临床试验将回答加入BTK抑制剂伊布替尼能否提高年轻可耐受患者的疗效，以及靶向药是否可取代ASCT的地位[28]。

3. 高龄或不适合移植患者的诱导治疗方案：对于老年一般情况较差的患者，NCCN指南优先推荐4个方案：BR、VRCAP、R-CHOP和利妥昔单抗+来那度胺（R2）。对于前三个方案，有多个前瞻性随机对照临床试验证实BR和VRCAP优于R-CHOP，但未比较BR和VRCAP。从横向数据看，BR方案的PFS时间最长，为3～4年，VRCAP的PFS时间约2年，而R-CHOP的PFS时间为1～2年，因此BR方案值得优先推荐[29]–[30]。R2方案的优先推荐证据来自一项小样本研究，共纳入38例初诊患者，ORR为92％，CR率为64％，5年PFS率和OS率达64％和77％[31]–[32]，优于BR、VRCAP、R-CHOP方案的横向数据。NCCN指南暂未将BTK抑制剂列入一线治疗中进行推荐，一项临床研究使用伊布替尼联合利妥昔单抗（IR）治疗50例老年不适合移植的初诊MCL患者，这些患者为非母细胞/多形性型，Ki-67<50％、最大肿瘤直径<10 cm，ORR 92％，CR率68％，3年PFS率87％，OS率94％[33]，疗效与BR及R2方案相当，因此，我们也纳入本版指南的一线推荐。考虑到我国多种BTK抑制剂的可及性，及不同BTK抑制剂疗效均较好，因此，在指南中并未限定伊布替尼联合利妥昔单抗，其他BTK抑制剂均可考虑。

4. 维持治疗：年轻MCL患者在一线接受大剂量Ara-C诱导方案及ASCT后接受利妥昔单抗维持治疗能明显改善生存[34]。一项欧洲多中心随机研究发现，一线应用R-CHOP方案的老年MCL患者应用利妥昔单抗维持治疗，PFS和OS均明显获益[35]。对于接受BR方案诱导化疗的老年初治患者，若达到CR，可不用利妥昔单抗维持，若仅达到部分缓解，利妥昔单抗维持可显著延长OS时间[36]，另一项随机研究结果则认为接受BR方案后继续应用利妥昔单抗维持无法使PFS和OS获益[37]。因此，指南推荐，MCL患者在接受R-CHOP或ASCT治疗后应进行至少2年的利妥昔单抗维持治疗，应用其他诱导方案的老年患者是否从利妥昔单抗维持治疗中获益，目前尚无足够证据支持。

除此之外，一项Ⅲ期临床研究尝试应用来那度胺维持治疗，ASCT后应用来那度胺维持者的PFS较不维持者提升，但不良反应也会增加，尤其是血液学毒性[38]，可作为不适合应用利妥昔单抗维持治疗患者的补充。老年MCL R2随机临床试验比较应用利妥昔单抗（R）维持和R2维持两组患者的预后，发现R2组患者PFS提升明显，但是OS无明显获益，且R2维持治疗的毒性明显高于R[39]，后续亚组分析发现，诱导治疗后达微小残留病（MRD）阴性的患者应用R2维持治疗优于R，但MRD阳性患者R2和R维持治疗结局相似[40]。可能对于治疗后达到深度缓解的患者，长期免疫调节维持治疗有助于其获得更持久的缓解，但结论有待进一步验证。另外，TRIANGLE研究也尝试探究利妥昔单抗联合BTK抑制剂维持治疗与利妥昔单抗单药相比能否使患者获益。

5. 复发难治（R/R）MCL的治疗：以BTK抑制剂为主的新药是R/R MCL的首选治疗方案，但不同患者的治疗反应和预后差别仍然很大。TP53突变的R/R患者应用伊布替尼治疗效果较差，中位PFS时间仅为4个月[41]。阿卡替尼临床试验亚组分析也发现，具有MIPI高危、侵袭性变型、Ki-67％>50％这些高危因素的R/R患者的总体反应率与其他患者相似，但反应持续时间较短[42]。泽布替尼在应用于R/R MCL患者时，虽然表现出更高的反应率和缓解深度，但对于TP53突变的患者疗效仍较差[43]。因此，在本版指南中也制定了R/R MCL的高危因素，包括：TP53突变/缺失、CDKN2A缺失、侵袭性变型、Ki-67％>50％，有别于NCCN等指南。

对于一线复发后伴有高危因素或二线治疗未达CR或BTK抑制剂治疗失败患者，指南首先推荐靶向CD19的CAR-T细胞治疗。国际、多中心、Ⅱ期ZUMA-2试验在68例R/R MCL患者中应用KTE-X19治疗，ORR为93％（CR率67％），57％患者在12个月的随访后仍处于缓解状态[17]。亚组分析中，母细胞样、TP53基因突变及高Ki-67的MCL患者在接受KTE-X19治疗后反应率与其他患者相似[17],[44]。另一款CAR-T细胞产品Liso-cel表现出更好的安全性，≥3级的细胞因子释放综合征发生率仅为3％，有效率较高（ORR 84％，CR率59％）。因此，抗CD19 CAR-T治疗是目前R/R高危MCL的较优选择（图1）。

**图1:**
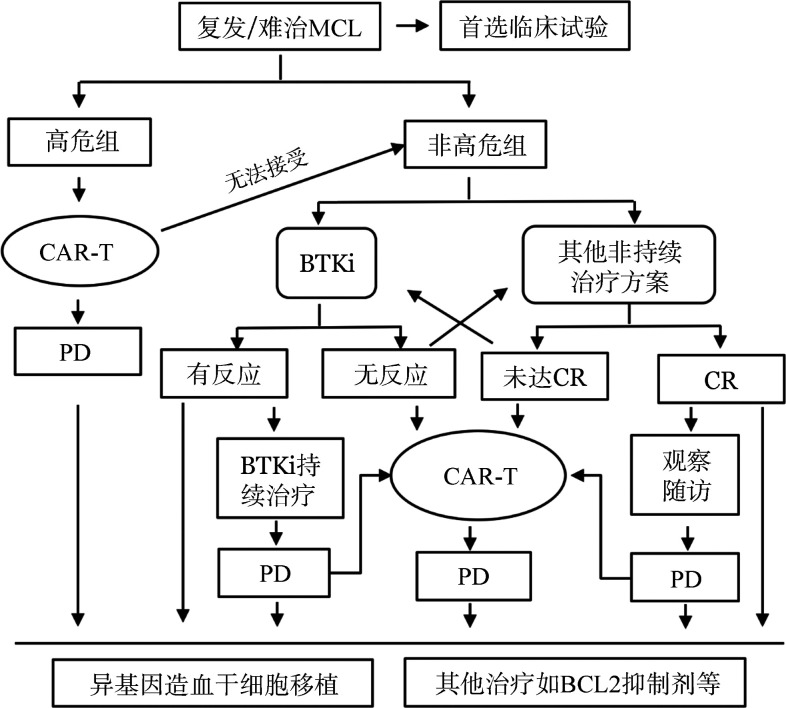
复发难治套细胞淋巴瘤（MCL）的治疗流程 注 CR：完全缓解；PD：疾病进展；BTKi：布鲁顿酪氨酸激酶抑制剂；CAR-T：嵌合抗原受体T细胞治疗

对于非高危组患者，在BTK抑制剂和化疗方案中如何选择，目前尚无标准答案，最近一项纳入261例年轻R/R MCL回顾性队列分析发现，R-BAC方案和伊布替尼在PFS上优于BR方案或其他方案，虽然在首次缓解到复发的时间大于24个月的患者中，伊布替尼和BR方案之间的差异无统计学意义，但对于24个月内复发的患者，伊布替尼在PFS和OS上均明显优于其他方案[45]。

多项临床试验证实单药BTK抑制剂治疗R/R MCL是安全有效的可选方案，总反应率为60％～80％，PFS时间为11～15个月[46]–[48]。Wang等[49]报道，R2方案治疗复发MCL，ORR达57％，CR率达36％，尤其在BTK抑制剂治疗失败或不耐受的患者中有较好的耐受性和有效性[50]。BR方案在R/R MCL中具有高度活性，在前瞻性研究中，ORR为70％～90％，中位PFS时间约为18个月[51]–[52]。R-BAC方案治疗R/R MCL则具有更高的有效率，2年PFS率达70％[53]，而且此方案在接受BTK抑制剂治疗失败的患者中也有较高的反应率（ORR 83％，CR率60％）[54]。这些数据表明，非高危患者首先推荐应用BTK抑制剂治疗或R2方案治疗，特别是对于一线强化诱导治疗难治或早期复发的患者，诱导缓解后，建议年轻且有合适全相合供者的患者进行减低剂量预处理的异基因造血干细胞移植（allo-HSCT）[55]（图1）。

CAR-T细胞和allo-HSCT的时机和顺序如何选择、新药时代下是否还需要行allo-HSCT是现在争论的热点问题。allo-HSCT的优势是长期随访数据和移植物抗淋巴瘤效应的明确证据，CAR-T细胞的优点是对耐药和活动性高危MCL具有明显的疗效和较低的长期毒性，allo-HSCT被一致认为是MCL的治愈性手段[17]，也可能可以克服TP53突变带来的不良预后影响。一项小系列研究（42例患者）表明，伴有TP53突变且接受allo-HSCT的R/R MCL患者与无TP53突变患者的生存结果大致相同[56]。但allo-HSCT后1年移植相关致死率达10％～20％[57]。来自美国移植和细胞治疗学会（ASTCT）、国际血液和骨髓移植研究中心（CIBMTR）和欧洲骨髓移植学会（EBMT）最新共识都认为，对于R/R MCL，尤其是BTK抑制剂治疗失败的患者，可首先选择CAR-T细胞治疗；如果CAR-T细胞治疗失败或无法进行，可选择allo-HSCT[58]。如果CAR-T细胞治疗3个月后，患者治疗有效但是未达到CR，预期远期预后较差，此时也可以选择allo-HSCT增加患者获得长期疾病控制的希望[58]。

其他新药临床试验也在R/R MCL患者中表现出较好的安全性和有效性，包括新一代非共价结合的BTK抑制剂如LOXO-305、PI3K抑制剂、BCL2抑制剂、ROR1偶联单抗、抗CD20/CD3双克隆抗体等，均处于临床研究阶段，虽然目前BTK抑制剂、免疫化疗或CAR-T细胞在R/R MCL患者中取得了较好的进展，但R/R MCL的整体预后仍较差，尤其是高危患者，因此我们仍推荐一线治疗后复发患者首选参加设计良好的临床试验。

五、展望

含大剂量Ara-C的方案诱导缓解后进行ASCT仍是目前可耐受MCL患者的标准治疗方案，BTK抑制剂、来那度胺、BCL2抑制剂等新药的出现，进一步提高了MCL的缓解率和生存时间。根据MCL的危险因素和遗传学异常进行个体化分层治疗，探索最为有效的药物组合及无细胞毒化疗方案的可行性和适合人群，是未来的方向。
